# Cracking the code of HBV persistence: cutting-edge approaches to targeting cccDNA in chronic hepatitis B with or without pyogenic liver Abscesses

**DOI:** 10.3389/fmed.2025.1504736

**Published:** 2025-03-17

**Authors:** Umar Saeed, Zahra Zahid Piracha, Mahmood Khan, Muhammad Nouman Tariq, Syed Shayan Gilani, Muhammad Raza, Rakshana Munusamy, Naveen Bose, Dilber Uzun Ozsahin, İlker Özşahin, Surya M. Nauli

**Affiliations:** ^1^Operational Research Center in Healthcare, Near East University, Nicosia, Türkiye; ^2^Department of Clinical and Biomedical Research Center (CBRC), Foundation University School of Health Sciences (FUSH), Foundation University Islamabad (FUI), Islamabad, Pakistan; ^3^Department of Medical Lab Technology, Al-Mizan Islamic International Medical College Trust (IIMCT) Complex, Riphah International University, Rawalpindi, Pakistan; ^4^International Center of Medical Sciences Research (ICMSR), Austin, TX, United States; ^5^International Center of Medical Sciences Research (ICMSR), Essex, United Kingdom; ^6^International Center of Medical Sciences Research (ICMSR), Islamabad, Pakistan; ^7^School of Rehabilitation, Kunming Medical University, Kunming, Yunnan, China; ^8^Akhtar Saeed Medical and Dental College, Lahore, Pakistan; ^9^Department of Medical Sciences, The Tamil Nadu Dr. M.G.R University, Chennai, India; ^10^Department of Medical Diagnostic Imaging, College of Health Sciences, University of Sharjah, Sharjah, United Arab Emirates; ^11^Research Institute for Medical and Health Sciences, University of Sharjah, Sharjah, United Arab Emirates; ^12^Department of Pharmacy, Chapman University, Irvine, CA, United States

**Keywords:** Chronic Hepatitis B Virus, hepatocellular carcinoma, molecular pathways, Hepatitis B, cccDNA

## Abstract

Chronic Hepatitis B Virus (HBV) infection remains a formidable global health challenge, driving severe liver complications such as hepatocellular carcinoma (HCC) and pyogenic liver abscesses (PLA). At the core of HBV persistence lies covalently closed circular DNA (cccDNA), a viral reservoir that fuels ongoing infection despite antiviral treatments. This review highlights molecular mechanisms governing cccDNA formation, maintenance, and clearance, spotlighting innovative therapeutic strategies to disrupt this key viral element. We explore cutting-edge approaches, including epigenetic modulation to silence cccDNA, RNA interference (RNAi) for viral RNA degradation, and CRISPR/Cas genome editing to excise cccDNA directly. Additionally, emerging antiviral therapies and immunotherapies, such as therapeutic vaccines and immune checkpoint inhibitors, offer new avenues for enhanced treatment efficacy. Special attention is given to the clinical complexities of managing HBV in patients with co-morbid conditions like HCC and PLA, emphasizing the necessity of a multidisciplinary approach. The interplay between antibacterial and antiviral therapies in PLA-associated HBV cases is critically examined to prevent treatment antagonism, ensuring optimal patient outcomes. Advanced therapeutic strategies, including nucleos(t)ide analogs, interferon therapy, and novel genomic interventions, are explored in both isolated HBV infection and PLA co-infections. Personalized regimens remain pivotal in enhancing therapeutic efficacy and long-term disease control. Current review advocates for a shift toward precision medicine, highlighting the critical need for interdisciplinary collaboration to bridge molecular discoveries with clinical innovations. Ultimately, these advancements promise to revolutionize the management of chronic HBV, paving the way for potential cures and improved patient outcomes.

## Introduction

Hepatitis B Virus (HBV) infection continues to present a monumental global health challenge, affecting over 250 million people worldwide. While chronic HBV infection is a leading cause of liver disease, it is also a precursor to severe complications such as hepatocellular carcinoma (HCC) and pyogenic liver abscesses (PLA). At the heart of this persistent infection is the covalently closed circular DNA (cccDNA), a resilient viral reservoir that resides in the nuclei of infected hepatocytes, ensuring the virus’s survival despite ongoing treatment. Current therapies as shown in Table 1, includes nucleos(t)ide analogs (NAs) and interferons (IFNs), effectively suppress HBV replication but fail to target cccDNA, requiring lifelong treatment. This limitation underscores the urgent need for innovative therapies that specifically eliminate cccDNA, a critical step in overcoming chronic HBV infection and improving outcomes for patients, particularly those suffering from HCC and PLA, where co-infection complicates management (1, 2).

**TABLE 1:** Current treatment for Hepatitis B Virus (HBV).

Treatment	Dosage	Duration	Mechanism	Notes/citations
Nucleos(t)ide analogs (NAs)	Varies per drug (e.g., Tenofovir 300 mg daily)	Lifelong treatment due to inability to clear cccDNA	Suppresses viral replication by inhibiting HBV DNA polymerase	(3–6)
Interferons (IFNs)	Peg-IFN-α2a: 180 μg/week	48 weeks	Stimulates immune response to control viral infection	Can lead to significant side effects, poor tolerance in some patients (7–9)
Entecavir	0.5 mg daily	Long-term	Inhibits HBV DNA replication	Often preferred for antiviral resistance (15, 16, 24)

Chronic Hepatitis B Virus (HBV) infection continues to pose significant challenges in clinical practice, particularly when complicated by the co-occurrence of hepatocellular carcinoma (HCC) and pyogenic liver abscess (PLA). This triad of conditions underscores a complex clinical scenario where effective management requires careful consideration of both viral and bacterial pathogens, as well as their interactions within the host environment. While HBV is a key contributor to the pathogenesis of HCC through its effects on viral replication, immune modulation, and tumorigenesis, PLA, primarily caused by bacterial infections, presents a distinct set of challenges related to immune suppression and exacerbation of HBV persistence. The need for a more nuanced understanding of how HBV, HCC, and PLA intersect is critical to developing targeted therapeutic strategies that address the unique needs of these patients. The complexity of managing HBV in the context of co-infection with HCC and PLA lies in the intricate interplay between viral persistence, immune responses, and bacterial pathogens. For patients with HBV-related HCC, controlling HBV replication is paramount to mitigating tumor progression and improving survival outcomes. However, the pathogenesis of HCC itself is multifactorial, involving not only the regulation of HBV replication but also disruptions in the cell cycle, chronic inflammation, and tumor cell interactions. Effective treatment strategies for HCC must consider these factors while maintaining control over HBV replication, often through a combination of antiviral therapies, immune checkpoint inhibitors, and targeted treatments like sorafenib. However, research on the most effective approaches to combine antiviral therapy with HCC treatment remains ongoing, with limited consensus on optimal regimens. In contrast, PLA, which primarily results from bacterial infections, introduces a different layer of complexity. The treatment of PLA requires urgent antibiotic intervention, yet this must be balanced with careful management of HBV infection, as bacterial infections can exacerbate HBV persistence and complicate antiviral therapy. While there is evidence suggesting that coordinated antibacterial and antiviral therapy can mitigate complications in these patients, there is still a lack of robust clinical data to guide treatment decisions for patients with both HBV and PLA.

At the molecular level, the persistence of HBV is largely driven by the presence of covalently closed circular DNA (cccDNA) in hepatocytes. This episomal form of viral DNA is highly stable and resistant to current antiviral therapies, making it a critical target for new treatment strategies, as shown in Table 2. The ability to clear or modify cccDNA remains a major barrier to achieving a functional cure for HBV, especially in patients with concurrent HCC or PLA. The persistence of cccDNA in hepatocytes contributes not only to chronic viral replication but also to immune evasion, which plays a pivotal role in the progression of liver diseases, including cirrhosis and liver cancer.

**TABLE 2:** New therapeutic approaches for Hepatitis B.

Therapy	Mechanism of action	Stage of development	Notes/citations
RNA interference (RNAi)	Targets and degrades HBV RNA transcripts	Preclinical to clinical	siRNAs targeting HBV RNA have shown reduction in viral replication (8, 9)
Small interfering RNA (siRNA)	Selectively degrades HBV RNA transcripts, reducing replication	Preclinical to clinical trials	siRNAs like ARC-520 have shown promise but face delivery challenges (29)
Short hairpin RNA (shRNA)	Silences HBV gene expression through RNA-induced silencing	Early-stage research	Still under investigation for efficient delivery and efficacy (28)
CRISPR/Cas systems	Directly targets and edits HBV cccDNA	Preclinical	Potential to permanently disrupt cccDNA but still faces challenges (32)
Epigenetic modulators (HDACi)	Modifies histone acetylation to suppress HBV cccDNA transcription	Preclinical	Histone deacetylase inhibitors show promise in reducing viral load (7)
DNA methylation modulators	Silences HBV cccDNA via DNA methylation	Preclinical	Promising findings on silencing of cccDNA, e.g., DNMT inhibitors (19–21)

In patients with HCC, cccDNA contributes to the chronic inflammatory milieu and the dysregulation of the host’s immune responses, which are key factors in tumorigenesis. Targeting cccDNA with novel interventions such as RNA interference (RNAi), CRISPR/Cas gene editing, and epigenetic therapies is an area of intense research. These therapies aim to disrupt the maintenance and replication of cccDNA, potentially reducing the viral load and improving the efficacy of concurrent cancer treatments. However, the relationship between cccDNA and bacterial infections in the context of PLA remains underexplored. The immune suppression caused by bacterial infections could enhance HBV persistence through a variety of mechanisms, including the reduction of antiviral immune responses, potentially complicating the eradication of cccDNA in these patients.

**FIGURE 1:**
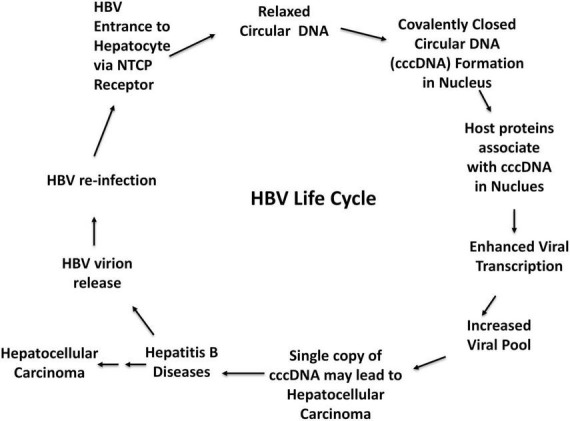
Hepatitis B Virus life cycle, risks for hepatocellular carcinoma and other liver complications.

**FIGURE 2:**
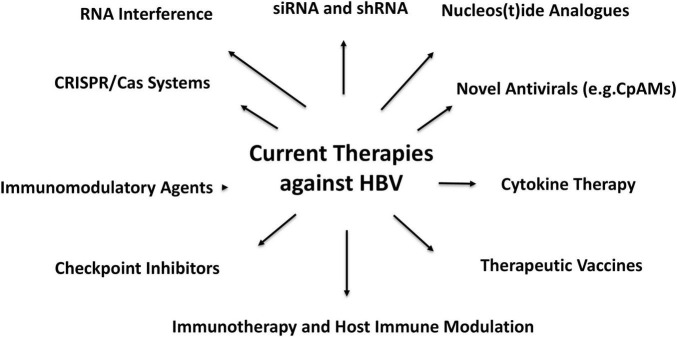
Current Hepatitis B Virus (HBV) therapeutics and hope for antiviral strategies against HBV.

Uncovering the complex regulatory mechanisms controlling cccDNA formation (Figures 1, 2), maintenance, and clearance is now recognized as essential to developing next-generation HBV therapies (3–6). In response, an expanding body of research is focusing on cutting-edge approaches to directly target cccDNA at multiple stages of its lifecycle, from modulating transcription to manipulating epigenetic pathways and leveraging immune-based therapies to disrupt its persistence.

A particularly exciting area of research is the modulation of host-cellular factors that govern cccDNA stability and transcription. Li et al. (7) demonstrated the potential of histone deacetylase inhibitors to effectively suppress cccDNA transcription, presenting a promising therapeutic strategy to reduce viral replication and mitigate the development of HCC (7). Similarly, Zhang et al. (8) explored the role of DNA methylation in cccDNA silencing, revealing that epigenetic interventions could be a powerful tool in the fight against HBV. Additionally, RNA interference (RNAi) has emerged as a groundbreaking technology, offering the ability to selectively target and degrade cccDNA-derived RNA. Liu et al. (9) developed small interfering RNAs (siRNAs) that specifically target HBV RNA transcripts, reducing viral replication and lowering the risk of HCC in preclinical models. These studies underscore the transformative potential of RNAi-based therapeutics in revolutionizing HBV treatment (8, 9).

Together, these novel approaches, from epigenetic modulation to RNA interference, are reshaping the landscape of HBV treatment, offering new hope for the eradication of cccDNA. With continued research, these innovations could pave the way for curative therapies that not only eliminate the viral reservoir but also provide a long-term solution for chronic HBV infection and its associated complications.

In this comprehensive study, we explore the intricate molecular mechanisms underpinning chronic HBV infection, with a particular focus on the critical role of cccDNA in sustaining HBV persistence. We examine various therapeutic strategies aimed at targeting cccDNA in patients with chronic HBV infection, whether or not they also present with PLA. The co-occurrence of PLA in HBV patients significantly complicates treatment strategies, highlighting the importance of understanding how these co-infections impact disease progression and the management of HBV-related liver complications. Additionally, we investigate the complex interplay between HBV infection, hepatocellular carcinoma (HCC), and PLA, all of which can exacerbate liver dysfunction and hinder effective treatment. This review provides insights into recent advancements in understanding how these conditions interact and how emerging therapeutic strategies are addressing the challenges posed by these interconnected diseases. In addition to reviewing the molecular pathways involved, we highlight promising therapeutic innovations targeting cccDNA, including cutting-edge approaches such as CRISPR/Cas9 gene editing, RNA interference (RNAi), and epigenetic modulation. These therapies show potential in disrupting cccDNA replication and mitigating viral persistence, offering new hope for more effective treatments. This review underscores the importance of personalized therapeutic approaches and the growing potential for curative strategies in managing chronic HBV infection and its associated complications.

Despite significant advances in the understanding of HBV pathogenesis, there remains a lack of consensus among clinicians and researchers regarding the most effective treatment regimens for HBV patients with co-existing HCC and PLA. The multifactorial nature of HCC and the bacterial etiology of PLA require individualized treatment approaches, yet there is insufficient clinical evidence to guide these decisions. The challenge lies in balancing the treatment of HBV infection with the management of bacterial infections and cancer, especially in terms of drug interactions, immune modulation, and potential adverse effects. Future research should prioritize elucidating the molecular mechanisms that govern the interactions between HBV, HCC, and PLA, with particular focus on how cccDNA contributes to these dynamics. Investigating the impact of immune modulation, such as the use of immune checkpoint inhibitors, in combination with antiviral therapies could offer valuable insights into improving clinical outcomes for these patients. Additionally, exploring the potential for combination therapies that target both HBV and bacterial infections could provide new avenues for managing co-infected patients, offering hope for more effective, integrated treatment strategies. Management of HBV infection in the context of HCC and PLA remains a critical clinical challenge, advancing our understanding of the molecular mechanisms involved, particularly those related to cccDNA, will be essential for developing more effective treatment strategies. The complex interactions between viral persistence, immune evasion, and bacterial co-infection necessitate a comprehensive approach that combines antiviral therapy with targeted cancer treatments and bacterial infection management. Only through continued research and interdisciplinary collaboration will we be able to identify optimal treatment regimens for these patients and, ultimately, improve patient outcomes in the face of chronic HBV infection complicated by HCC and PLA.

### Targeting cccDNA maintenance: innovative strategies in HBV therapeutics

The persistence of covalently closed circular DNA (cccDNA) is a cornerstone in hepatitis B virus (HBV) infection, facilitating chronic infection and rendering eradication strategies highly challenging. as the stable genetic reservoir of the virus, cccDNA ensures continuous viral replication and gene expression even amidst antiviral treatments and immune responses. This unique characteristic underscores the difficulty in developing curative therapies for chronic HBV infection. Therefore, a deeper understanding of the molecular mechanisms governing cccDNA formation, maintenance, and regulation is vital to developing groundbreaking therapeutic approaches aimed at eliminating HBV.

### Molecular mechanisms of cccDNA formation and stability

The formation of cccDNA begins when relaxed circular DNA (rcDNA) is converted into the covalently closed form, a crucial step facilitated by host DNA repair machinery. This transformation relies heavily on host enzymes such as DNA ligases, topoisomerases, and tyrosyl-DNA phosphodiesterase 2 (TDP2), which repair DNA breaks induced by the viral replication process. Among these, DNA ligase I and topoisomerase I are essential players in cccDNA formation. Cui et al. (10) demonstrated that inhibiting these enzymes significantly reduces cccDNA formation *in vitro*, suggesting that small molecule inhibitors targeting these enzymes could disrupt the viral lifecycle and mitigate chronic HBV infection (11). Interestingly, these enzymes also play a pivotal role in regulating the stability and transcription of cccDNA, offering new avenues for multi-targeted therapies aimed at both formation and functional regulation. Synthetic biology approaches are also gaining traction, exploring ways to engineer host enzymes that resist viral replication, potentially decreasing cccDNA formation and promoting viral clearance.

### Parvulin 14 and parvulin 17: key regulators of cccDNA

Previously we have highlighted a novel and critical role for Par14 and Par17, members of the parvulin family of peptidyl-prolyl isomerases, in regulating cccDNA levels in HBV-infected cells. These enzymes catalyze the cis-trans isomerization of proline residues, influencing protein folding and function. Par14 and Par17 have been shown to bind directly to cccDNA, as well as to the viral core protein (HBc) and HBX, both of which are vital for the HBV lifecycle. By enhancing cccDNA stability, these parvulins play a significant role in maintaining its transcriptionally active state, thereby facilitating ongoing viral replication. We demonstrated that inhibiting PIN4, the gene encoding both Par14 and Par17, leads to a dramatic reduction in cccDNA levels, presenting PIN4 inhibition as a promising strategy for destabilizing the viral genome and suppressing HBV replication. Targeting Par14 and Par17 could disrupt critical steps in the HBV lifecycle, offering an innovative antiviral strategy that disrupts both viral replication and the maintenance of cccDNA reservoirs.

### Epigenetic modulation and host factors: key therapeutic approaches

Epigenetic regulation of covalently closed circular DNA (cccDNA) plays a crucial role in its persistence within infected hepatocytes, making it a key target for therapeutic intervention. Modulating epigenetic marks, such as histone acetylation and DNA methylation, has emerged as a promising strategy to destabilize cccDNA and reduce its transcriptional activity, ultimately curbing HBV replication (12–14).

The epigenetic regulation of cccDNA is central to its persistence in infected cells. Histone modifications such as acetylation and methylation play a crucial role in the transcriptional activity of cccDNA. Histone deacetylase inhibitors (HDACi) like vorinostat have been shown to alter the chromatin structure of cccDNA, suppressing its transcription and, consequently, HBV replication. Zhang et al. (15) and Lucifora et al. (3) demonstrated that HDACi treatment significantly reduces cccDNA transcription, providing a promising approach to curtailing viral replication and progression toward hepatocellular carcinoma (HCC) (3, 15, 16). In addition to histone modifications, DNA methylation has emerged as a critical mechanism for silencing cccDNA. Tang et al. (17) demonstrated that methylation of cccDNA can effectively silence its transcription, thereby reducing viral replication (17). Recent research has also uncovered that cccDNA undergoes dynamic methylation changes in response to antiviral treatment, which could potentially reprogram the epigenetic landscape of cccDNA, offering a novel strategy for curative therapies by inducing permanent silencing of the virus.

Beyond histone acetylation, DNA methylation has also been identified as a critical epigenetic modification influencing cccDNA stability and transcriptional activity. Pharmacological agents that target DNA methyltransferases (DNMTs) have shown potential in altering the DNA methylation patterns associated with cccDNA, thus influencing its transcriptional regulation. The exploration of epigenetic modulators aimed at reprogramming the epigenetic landscape of cccDNA presents an intriguing and novel therapeutic approach for chronic HBV infection (18).

Guo et al. (19) demonstrated that increasing methylation of cccDNA using DNA methyltransferase inhibitors (DNMTis) like 5-azacytidine could lead to the transcriptional silencing of HBV (19). This strategy was further supported by Liu et al. (20), who showed that pharmacological modulation of DNA methylation could effectively silence cccDNA transcription, offering a potential therapeutic avenue for chronic HBV (20). More recently, Pollicino et al. (21) confirmed the role of DNMT inhibitors in altering cccDNA methylation patterns, leading to a reduction in viral replication and the promotion of HBV-infected cell clearance (21). The development of such DNA methylation-based therapies holds great promise for reducing HBV persistence and advancing toward a functional cure. Some of the novel therapeutic options targeting epigenetic modifications in HBV are discussed in Figure 2, highlighting emerging strategies for reprogramming cccDNA epigenetics.

Histone deacetylase inhibitors (HDACi) have been identified as potential candidates for disrupting cccDNA persistence by modulating histone acetylation. By inhibiting histone deacetylases, HDACi compounds promote histone hyperacetylation, which leads to transcriptional repression of cccDNA, thereby diminishing viral replication. Preclinical studies have demonstrated the efficacy of HDACi in reducing both cccDNA levels and viral activity in hepatocytes, suggesting their potential as therapeutic agents for HBV infection. For example, Li et al. (7) found that HDAC inhibitors significantly reduced cccDNA transcription and viral load (7). Similarly, studies by Liang et al. (22) and Wang et al. (23) demonstrated that HDACi treatment led to decreased viral replication and enhanced destabilization of cccDNA in HBV-infected cells (22, 23). Zhong et al. (18) emphasized the role of HDACi in suppressing HBV transcription through the alteration of the chromatin structure of cccDNA. Specific HDAC inhibitors, such as entinostat and panobinostat, have shown effectiveness in preclinical models, significantly reducing both cccDNA transcription and viral load. These findings underscore the potential of HDACi as promising therapeutics in the fight against chronic HBV infection.

### Targeting DNA repair pathways and immune surveillance

Host DNA repair pathways, particularly the cGAS-STING immune sensor pathway, also play a critical role in cccDNA maintenance. The cGAS-STING pathway, an essential component of the innate immune system’s response to DNA damage, has been implicated in recognizing and targeting viral DNA intermediates, including cccDNA. By modulating this immune surveillance system, it may be possible to enhance the immune system’s ability to clear HBV-infected cells, providing a promising immunotherapeutic strategy for combating HBV.

Moreover, TDP2 is another key player in the repair process. Levrero et al. (24) demonstrated that inhibiting TDP2 disrupts the repair process and prevents cccDNA formation, further highlighting the therapeutic potential of targeting host DNA repair machinery to reduce cccDNA levels and eliminate HBV reservoirs (10). Combining TDP2 inhibition with other DNA-damaging agents could offer synergistic effects, amplifying the suppression of HBV replication and promoting viral clearance.

### RNA interference and CRISPR/Cas9: emerging therapies for cccDNA elimination

RNA interference (RNAi) technology has also shown considerable promise in targeting cccDNA-derived transcripts, effectively reducing viral replication and cccDNA levels. Liu et al. (9) demonstrated that small interfering RNAs (siRNAs) could selectively degrade HBV RNA, leading to a significant reduction in HBV replication and HCC risk in preclinical models (9). RNAi technologies are rapidly evolving, with researchers working on improving delivery methods and selectivity for viral RNA, potentially allowing for sustained suppression of cccDNA.

In parallel, CRISPR/Cas9 gene-editing technology has revolutionized the ability to target specific DNA sequences, including cccDNA. Recent studies have demonstrated that CRISPR/Cas9 can directly edit cccDNA, leading to its degradation and elimination from infected cells, presenting the possibility of a functional cure for HBV. This innovative technology holds the potential to precisely target and excise cccDNA from the host genome, without causing collateral damage to host cells, thus paving the way for safer and more effective treatments.

### The road to a functional cure for HBV

These groundbreaking strategies offer a multifaceted approach to eliminating cccDNA, a major obstacle in achieving a cure for chronic HBV infection. Whether through epigenetic modulation, host-directed therapeutics, RNA interference, or gene-editing technologies like CRISPR, the progress in this field offers renewed hope for more effective treatments and better outcomes for patients with HBV-related liver diseases. The potential to destabilize cccDNA, reduce HBV replication, and ultimately eliminate the viral reservoir could usher in a new era of HBV therapies, moving us closer to a functional cure for this challenging infection.

### Small molecules targeting epigenetic readers

The recognition of specific histone and DNA modifications by epigenetic reader proteins is crucial for regulating chromatin structure and gene expression. Targeting these epigenetic readers with small molecules offers a promising strategy to modulate the epigenetic state of cccDNA and influence its transcriptional activity. By disrupting the binding of epigenetic readers to modified histones or DNA, these small molecules can precisely control cccDNA function, potentially leading to the development of novel therapeutic approaches for HBV infection (25, 26).

The exploration of therapies targeting the epigenetic regulation of cccDNA is an active and rapidly advancing field, with important implications for future HBV treatment strategies. Current research is focused on uncovering the molecular mechanisms that govern cccDNA’s epigenetic regulation and identifying new therapeutic agents capable of modulating its transcriptional activity and persistence in infected hepatocytes. These ongoing efforts highlight the potential of targeting cccDNA epigenetics through histone modifications, DNA methylation, and disruption of epigenetic reader proteins as a promising avenue for innovative HBV therapies. Continued research is essential to fully understand the mechanisms behind cccDNA epigenetic regulation and to translate these insights into effective clinical treatments for chronic HBV infection (13, 14, 16, 27).

### RNA interference

RNA interference (RNAi) has become an effective strategy for targeting HBV transcripts, promoting the degradation of viral RNA and suppressing viral replication. This technique uses small interfering RNA (siRNA) or short hairpin RNA (shRNA) to specifically target HBV RNA, facilitating its degradation via the RNA-induced silencing complex (RISC) (28).

#### siRNA and shRNA

Using siRNA and shRNA to target HBV RNA has shown promise in reducing viral RNA levels. Wooddell et al. (29) demonstrated that siRNAs targeting HBV RNA resulted in a significant reduction in both HBV RNA and DNA in infected hepatocytes, underscoring the potential of RNAi-based therapies in reducing viral replication and persistence (29).

RNA interference therapeutics, such as ARC-520, have shown initial success in lowering HBV RNA levels in clinical trials. However, challenges related to delivery and off-target effects remain obstacles to broader application. While ARC-520 has demonstrated potential, further refinement is necessary to improve its safety and efficacy in clinical settings (30). Ongoing developments in RNAi and gene-editing technologies offer opportunities for more effective targeting of cccDNA, with newer compounds addressing delivery challenges and off-target effects (30).

Additionally, other RNAi-based strategies are being explored. Zhang et al. (31) developed an innovative siRNA delivery system that enhances siRNA stability and cellular uptake, using nanoparticles to protect the siRNAs from degradation and improve their efficiency in targeting HBV RNA in hepatocytes (31).

RNA interference shows significant promise as a treatment for HBV infection. Continued research is essential to overcome current limitations and translate these findings into clinical practice. Combining RNAi with gene editing and epigenetic modulation may provide an effective, multi-faceted approach for achieving long-term viral suppression and potentially curing HBV infection.

### CRISPR/Cas systems

The CRISPR/Cas systems, notably CRISPR/Cas9 and CRISPR/Cas12a, have emerged as groundbreaking tools for directly targeting and cleaving hepatitis B virus (HBV) covalently closed circular DNA (cccDNA). By precisely designing these systems to target HBV cccDNA, researchers aim to either eliminate or mutate the viral genome, thereby inhibiting replication and significantly reducing viral load (33).

The application of CRISPR/Cas9 to HBV treatment has yielded promising outcomes. Seeger and Sohn (34) provided compelling evidence that CRISPR/Cas9 can induce double-strand breaks in HBV cccDNA, leading to its degradation. This pivotal study established the proof-of-concept for employing CRISPR technology to disrupt HBV cccDNA, laying a strong foundation for subsequent investigations in this field (34).

Expanding upon this innovative approach, Ramanan et al. (35) investigated the utility of CRISPR/Cas12a for targeting and disrupting HBV cccDNA. Their findings underscored the remarkable specificity of CRISPR/Cas12a in cleaving cccDNA, with notably reduced off-target effects compared to CRISPR/Cas9. This heightened specificity is particularly critical for minimizing potential collateral damage to the host genome, thereby enhancing the safety and therapeutic viability of CRISPR-based interventions (35). CRISPR/Cas-based technologies underscore their transformative potential in HBV treatment, marking a significant leap toward novel and effective therapeutic strategies.

Recent advancements have propelled the refinement of CRISPR technology for hepatitis B virus (HBV) treatment, introducing novel approaches with enhanced precision and efficacy. For instance, Liu et al. (36) developed an optimized CRISPR/Cas9 system with superior targeting accuracy, significantly minimizing off-target effects. This innovation represents a critical step toward improving the safety profile of CRISPR-based therapies. Similarly, Yin et al. (37) demonstrated the effective application of CRISPR/Cas12a in a preclinical model, achieving robust disruption of HBV cccDNA and substantial suppression of viral replication (36, 37).

Beyond the direct cleavage of cccDNA, the therapeutic potential of CRISPR/Cas systems extends to synergistic strategies that amplify their efficacy. Researchers are exploring innovative combinations of CRISPR technology with complementary modalities. For example, Chen et al. (38) investigated the integration of CRISPR/Cas9 with RNA interference (RNAi), achieving a synergistic reduction in HBV replication while enhancing cccDNA degradation. This combined approach underscores the versatility of CRISPR systems in addressing complex viral mechanisms (38).

The transformative promise of CRISPR/Cas systems in HBV therapy lies in their adaptability and precision. Continued research and meticulous optimization are imperative to unlock their full potential in clinical applications. By integrating CRISPR technology with other therapeutic strategies, a multifaceted approach could emerge, offering comprehensive solutions for eradicating HBV infection and achieving durable viral suppression. These advancements mark a significant stride toward realizing a curative paradigm for HBV.

### Emerging antiviral agents and immunotherapeutic interventions: disrupting cccDNA persistence

In addition to advancements in targeting the epigenetic regulation of cccDNA, innovative antiviral agents and cutting-edge immunotherapeutic interventions are emerging as promising strategies to disrupt cccDNA persistence and bolster immune-mediated clearance of hepatitis B virus (HBV) infection. These developments represent a crucial step forward in addressing the chronic nature of HBV, offering new avenues for achieving sustained viral suppression and potentially curative outcomes (39).

#### Nucleos(t)ide analogs

Nucleos(t)ide analogs (NAs) have long served as a cornerstone in the management of hepatitis B virus (HBV) infection, primarily exerting their antiviral effects by inhibiting reverse transcriptase activity and viral DNA synthesis (40). While NAs are highly effective in suppressing viral replication and lowering circulating viral loads, their direct impact on cccDNA levels within infected hepatocytes remains limited due to their inability to target cccDNA directly. Nevertheless, prolonged use of NAs has been associated with a gradual reduction in intracellular cccDNA reservoirs, underscoring their potential contribution to cccDNA decay over extended treatment durations (41).

Key NAs such as entecavir, tenofovir, and lamivudine function by inhibiting HBV polymerase, thereby reducing the production of relaxed circular DNA (rcDNA), a precursor to cccDNA. Among these agents, tenofovir and entecavir have demonstrated superior efficacy in suppressing HBV replication, effectively curtailing the replenishment of cccDNA over long-term therapy (42). Despite their limitations in directly targeting cccDNA, NAs remain an indispensable element of HBV treatment due to their ability to achieve sustained viral suppression and indirectly contribute to cccDNA reduction over time.

As the field advances, the development of novel antivirals offers the promise of complementing and enhancing the efficacy of existing NA-based therapies, providing a multifaceted approach to eradicating HBV infection.

The development of novel agents that specifically inhibit the formation or function of covalently closed circular DNA (cccDNA) represents a highly promising frontier in HBV research. Among these, core protein allosteric modulators (CpAMs) have emerged as a groundbreaking class of antivirals. By disrupting the assembly of relaxed circular DNA (rcDNA) into cccDNA, CpAMs effectively reduce cccDNA levels within HBV-infected cells. Recent investigations by Guo and Guo (43) have highlighted the potential of CpAMs in preventing cccDNA formation, reporting significant reductions in both cccDNA levels and viral replication. These findings underscore the critical role CpAMs could play in future HBV therapeutic strategies by directly targeting and interfering with the cccDNA lifecycle (43).

#### RNA interference therapeutics

The advent of RNA interference (RNAi) therapeutics has unveiled innovative opportunities for directly targeting cccDNA transcripts within HBV-infected hepatocytes. By employing small RNA molecules to selectively degrade cccDNA-derived RNA, RNAi offers a compelling approach to reducing transcriptional activity and suppressing viral antigen production. In a landmark study, Wooddell et al. (29) demonstrated the efficacy of siRNAs targeting HBV transcripts, achieving significant reductions in HBV RNA and DNA levels in infected hepatocytes. Further preclinical investigations have validated the therapeutic potential of RNAi agents, showing robust suppression of cccDNA-derived RNA and viral protein expression, emphasizing their pivotal role in addressing cccDNA persistence (29).

Clinical trials involving RNAi therapeutics, such as ARC-520, have shown promising outcomes, albeit with notable challenges related to delivery efficiency and off-target effects. For instance, Liang et al. (44) reported the successful application of RNAi-based therapies in animal models, achieving substantial reductions in HBV replication while highlighting the critical need for optimization in delivery mechanisms to enhance specificity and minimize collateral effects (44). More recently, Zhang et al. (31) introduced a groundbreaking siRNA delivery system that significantly improves the stability and cellular uptake of siRNAs in hepatocytes, thereby enhancing the precision and efficiency of HBV transcript targeting (45).

Together, these advances in RNAi therapeutics and complementary immunotherapeutic interventions present a transformative potential for disrupting cccDNA persistence and achieving sustained viral suppression. Continued efforts in research and development are imperative to address existing limitations and translate these promising preclinical breakthroughs into effective clinical therapies for chronic HBV infection.

### Immunotherapy and host immune modulation

Enhancing the host immune response to effectively clear HBV-infected cells harboring cccDNA represents a promising and innovative strategy for combating chronic HBV infection. Immunotherapeutic approaches focus on strengthening the immune system’s capacity to detect and eliminate infected hepatocytes, thereby addressing one of the key barriers to reducing cccDNA persistence. These strategies hold immense potential to complement existing therapies by targeting the underlying reservoirs of HBV infection, paving the way for more comprehensive and durable treatment outcomes (46).

#### Checkpoint Inhibitors

Checkpoint inhibitors, such as nivolumab and pembrolizumab, which specifically target the programmed death-1 (PD-1) pathway, have demonstrated significant potential in reinvigorating T-cell responses in patients with hepatocellular carcinoma (HCC) associated with chronic hepatitis B virus (HBV) infection. These immune checkpoint inhibitors work by blocking the interaction between PD-1 on T cells and its ligands, PD-L1 and PD-L2, which are often upregulated in the tumor microenvironment and in HBV-infected liver cells. As a result, these inhibitors can restore T-cell function, mitigate the exhaustion of T cells, and improve the body’s immune response against the virus. In a pivotal study, Gane et al. (47) highlighted the therapeutic potential of PD-1 inhibitors in the context of HBV infection, demonstrating that they could facilitate the immune system’s ability to combat HBV by reversing T-cell exhaustion. This reversal not only enhances immune surveillance but also contributes to the clearance of HBV-infected hepatocytes, including those harboring the covalently closed circular DNA (cccDNA), which serves as a major barrier to viral eradication. As a result, PD-1 inhibitors are being actively investigated in clinical trials to assess their safety and effectiveness in broader HBV-infected populations, with the goal of potentially transforming the therapeutic landscape for HBV-associated HCC. This approach underscores the growing interest in leveraging immune checkpoint blockade as a novel strategy for treating HBV-related liver diseases, including HCC (47).

#### Advancements in Therapeutic Vaccines for HBV: Targeting cccDNA and Viral Proteins

Chronic Hepatitis B Virus (HBV) infection continues to be a significant global health concern, driven in large part by the persistence of covalently closed circular DNA (cccDNA) in hepatocytes. The development of effective therapeutic vaccines capable of targeting cccDNA and the viral proteins HBx and HBc is emerging as a promising strategy to overcome the challenge of HBV eradication. These proteins play a pivotal role in the formation and maintenance of cccDNA, making them ideal candidates for targeted vaccine design. By focusing on these key elements of HBV’s life cycle, novel vaccine approaches aim to enhance immune responses that specifically eliminate HBV-infected hepatocytes.

HBx and HBc proteins are central to the persistence of HBV within the liver. HBx contributes to viral replication and enhances the stability of cccDNA by interacting with host cell machinery, while HBc serves as the core protein of the virus, encapsulating the viral genome and facilitating its replication. The interaction between these proteins and cccDNA is a major obstacle in achieving a cure for chronic HBV infection. By targeting these proteins, vaccines could disrupt HBV’s ability to maintain cccDNA in infected hepatocytes.

Recent immunoinformatics approaches have significantly advanced our understanding of how to design vaccines targeting these critical viral components. Previously, we have identified conserved epitopes in both HBx and HBc proteins that can serve as targets for effective vaccine development. These epitopes, derived from global consensus sequences of HBx and HBc, represent potential sites for peptide vaccine design, RNA interference strategies, and the creation of site-specific anti-HBV agents. The conservation of specific residues across all HBV genotypes (A-J) further enhances the potential of these epitopes for universal vaccine development. Notably, residues such as 52H to 59P in HBx and 137C in HBx are critical for HBV replication and transactivation, respectively, and are conserved across all genotypes, making them ideal targets for immune-based therapies (48).

Furthermore, MHC-I and MHC-II epitopes identified for both HBx and HBc proteins are crucial for the development of T-cell-based vaccines. Epitopes like X-M2, X-M5, X-M8, X-M11, X-M12, and C-M1, C-M2, C-M4, C-M6-11 are highly conserved and play critical roles in the presentation of HBV antigens to T-cells. These epitopes could significantly enhance T-cell-mediated immunity against HBV-infected hepatocytes, potentially leading to more effective viral clearance. Additionally, B-cell-based vaccines targeting epitopes such as X-B2 and X-B4 for HBx and C-B6 and C-B7 for HBc have shown promise in stimulating antibody-mediated immune responses, which could reduce viral load and interfere with viral replication (48, 49).

In addition to targeting HBx and HBc, the incorporation of dendritic cells engineered to present HBV antigens has emerged as a promising strategy to boost immune responses. Research by Bertoletti et al. (49) demonstrated that these engineered dendritic cells could enhance immune control over HBV replication, thereby facilitating the reduction of cccDNA persistence in infected hepatocytes. This approach, alongside the development of mRNA vaccines, which have gained widespread attention due to their success in COVID-19, is now being explored for HBV. mRNA vaccines allow for the direct delivery of genetic material encoding HBV antigens into cells, stimulating both humoral and cell-mediated immunity. Early trials with mRNA-based vaccines for HBV have shown robust immune activation and provide hope for a more targeted and effective vaccine platform (49).

Furthermore, combination therapies are being explored to enhance vaccine efficacy. These combinations include the use of immune checkpoint inhibitors to counteract immune exhaustion, a common issue in chronic HBV infection. By blocking immune checkpoints like PD-1, these inhibitors can restore the function of exhausted T-cells, enabling a stronger immune response to HBV-infected hepatocytes. The synergistic effect of combining vaccines with immune checkpoint inhibitors could significantly enhance the immune clearance of HBV, including its cccDNA reservoir (48, 49).

In addition to vaccination approaches, the use of CRISPR/Cas9-based genome-editing techniques to directly target and excise cccDNA offers a groundbreaking avenue for HBV cure strategies. Recent research has shown that CRISPR/Cas9 can effectively edit the HBV genome in infected cells, excising cccDNA or inducing mutations that prevent its replication. While still in the pre-clinical stages, this approach holds great promise as a direct method to eliminate the viral reservoir, providing a potential permanent solution to HBV infection (49, 50).

The future of HBV vaccination lies in the integration of these cutting-edge strategies. By targeting both viral proteins (HBx and HBc) and leveraging innovative platforms such as mRNA vaccines, dendritic cell-based vaccines, CRISPR/Cas9 genome editing, and immune checkpoint inhibitors, researchers are on the cusp of developing a comprehensive, multi-pronged approach to HBV treatment. This integrated strategy aims not only to reduce viral load but also to tackle the persistent cccDNA reservoir, a major barrier to a functional cure.

As clinical trials for these novel vaccine candidates progress, the potential for achieving sustained virological control and functional cure of chronic HBV infection continues to increase. The development of universal vaccines targeting conserved epitopes across HBV genotypes, combined with personalized therapeutic strategies, offers the promise of more effective and durable solutions for individuals suffering from chronic HBV infection. The combination of therapeutic vaccines targeting HBx and HBc proteins, novel vaccine platforms like mRNA, and genome-editing technologies such as CRISPR/Cas9 represents a paradigm shift in the fight against chronic HBV. With continued research, the translation of these innovations into clinical practice may lead to more effective, targeted, and personalized treatment options, ultimately paving the way for the eradication of HBV as a global health threat.

#### Cytokine therapy

Cytokine therapy, particularly the use of interferons (IFNs), offers a compelling approach to enhancing antiviral immune responses in the context of chronic HBV infection. Interferons, including IFN-α and IFN-λ, are known for their potent immunomodulatory properties, which can significantly bolster the body’s natural defenses against viral infections. Lanford et al. (50) explored the therapeutic potential of IFN-α and IFN-λ, revealing that these cytokines not only stimulate robust antiviral immune responses but also contribute to a marked reduction in cccDNA levels within infected hepatocytes. The mechanism underlying this effect is tied to the ability of interferons to activate the host’s innate immune system, thereby creating a more hostile environment for the virus. This activation enhances the overall antiviral state in the liver, which in turn facilitates the degradation of cccDNA and inhibits HBV replication. Through these actions, interferon therapies provide a dual benefit, strengthening the body’s immune surveillance while directly targeting the viral reservoir. As a result, interferons are emerging as a valuable therapeutic tool in the fight against chronic HBV infection, offering new hope for reducing the persistent presence of cccDNA in liver cells and potentially advancing toward the goal of a functional cure (50).

#### Immunomodulatory agents

Immunomodulatory agents, which target both innate and adaptive immune pathways, are emerging as promising tools in the fight against chronic HBV infection, particularly for their potential to disrupt the persistence of cccDNA and enhance immune surveillance of HBV-infected hepatocytes. Zoulim et al. (51) highlighted the ability of these agents to modulate the function of immune cells, thereby promoting robust antiviral immune responses. By fine-tuning the host’s immune system, these strategies can help to facilitate the immune-mediated clearance of cccDNA reservoirs, which are a major barrier to eradicating the virus. The exploration of immunomodulatory agents represents an exciting and dynamic frontier in the development of therapies that may eventually overcome the challenge of cccDNA persistence, which is critical for achieving a functional cure for chronic HBV infection.

The convergence of cutting-edge antiviral agents with immunotherapeutic interventions is rapidly becoming a cornerstone in the search for curative strategies for chronic HBV. As research continues to elucidate the intricate interactions between viral persistence and host immune responses, there is increasing optimism that combinatorial treatment regimens, utilizing the synergistic effects of multiple therapeutic modalities, could offer novel avenues for achieving sustained virological control and long-term remission. For instance, Zhang et al. (52) proposed that combining immune checkpoint inhibitors with therapeutic vaccines could significantly enhance the overall efficacy of HBV treatment. Such combinatorial approaches hold the potential to provide a more comprehensive and targeted strategy for eradicating HBV, ultimately offering the prospect of durable clinical remission and a functional cure. This emerging paradigm underscores the importance of integrating diverse therapeutic approaches to achieve the ultimate goal of eliminating HBV and its associated liver disease.

#### HCC and PLA interlinked contexts

The management of chronic Hepatitis B Virus (HBV) infection becomes increasingly intricate in the settings of hepatocellular carcinoma (HCC) and pyogenic liver abscess (PLA). These clinical conditions present unique challenges that require tailored therapeutic strategies to address both the persistence of HBV and the complex complications associated with these diseases (53–56).

### Hepatocellular carcinoma (HCC)

The integration of antiviral therapies with conventional HCC treatments has shown promising potential in managing both tumor growth and HBV replication. Combination therapies, such as the pairing of antiviral agents with targeted treatments like sorafenib, have demonstrated synergistic effects, improving clinical outcomes. Bruix et al. (57) emphasized that the combination of sorafenib with antiviral therapies not only suppressed tumor progression but also reduced HBV replication, leading to enhanced patient outcomes. In addition, the exploration of immune checkpoint inhibitors (ICIs), such as nivolumab, in conjunction with antiviral treatments has gained traction in HCC management. Gane et al. (47) reported that PD-1 inhibitors like nivolumab could reinstate T-cell function in patients with HBV-associated HCC, thus providing a promising approach for dual-targeting both the tumor and the virus. This dual therapy concept offers the potential for enhanced control over both cancer and chronic HBV infection, a crucial factor in improving survival rates in these complex patients.

The tumor microenvironment (TME) in HCC plays a pivotal role in enabling immune evasion and maintaining HBV persistence. Modulating the TME to promote antiviral immune responses represents a potential strategy for improving therapeutic outcomes. Recent studies have concentrated on altering the TME to enhance immune surveillance and disrupt HBV persistence, with promising results. For example, Reig et al. (58) demonstrated that immune checkpoint inhibitors could reshape the TME, not only improving antiviral immune responses but also reducing the persistence of cccDNA within malignant tissues. By targeting specific immune checkpoints or cellular pathways within the TME, such therapies aim to create a more favorable environment for immune-mediated clearance of both the virus and tumor cells, offering a dual therapeutic benefit in HBV-related HCC.

This evolving landscape of combinatorial therapies and TME modulation underscores the increasing complexity and sophistication of treatment strategies in the dual management of HBV and HCC, pointing toward more integrated and potentially curative approaches for patients facing these intertwined challenges.

### Managing chronic hepatitis B in the presence of pyogenic liver abscesses (PLAs)

Chronic Hepatitis B Virus (HBV) infection presents a formidable clinical challenge, particularly when complicated by pyogenic liver abscesses (PLAs). These bacterial infections, if left untreated, can accelerate liver damage, compromise immune responses, and exacerbate HBV persistence. An integrated, multidisciplinary strategy is required to manage both infections simultaneously while preventing adverse interactions between antibacterial and antiviral therapies.

### Critical considerations in pLA-complicated HBV infection

#### Prompt and aggressive antibacterial therapy

Early initiation of broad-spectrum antibiotics is paramount in managing PLA-associated bacterial infections. The choice of antibiotics should be guided by microbial culture and sensitivity testing to ensure optimal pathogen eradication. Immunosuppressed patients, particularly those with chronic HBV, may require adjunct antifungal therapy to mitigate the risk of disseminated fungal infections. Aggressive antibacterial therapy while maintaining HBV control is necessary (59). They demonstrated that precise coordination between antibiotic administration and antiviral therapy could minimize immune disruption and liver inflammation, thus reducing the risk of exacerbating chronic liver disease.

#### Drainage and surgical interventions

The size, number, and location of liver abscesses dictate the necessity of percutaneous drainage or surgical intervention. Imaging-guided percutaneous drainage remains the preferred approach, offering a minimally invasive solution for resolving abscesses while maintaining liver function. In cases of refractory or multiloculated abscesses, surgical intervention may be required to prevent widespread hepatic necrosis and systemic sepsis.

#### Optimization of antiviral therapy

Sustained suppression of HBV replication is crucial in patients with concurrent PLA. The selection of antiviral agents must take into account potential pharmacokinetic interactions with antibiotics. First-line nucleos(t)ide analogs (NAs) such as entecavir (ETV) and tenofovir (TDF) remain the cornerstone of HBV management, effectively inhibiting viral replication with minimal risk of resistance. Maintaining antiviral therapy during PLA treatment is critical, noting that disruptions could lead to HBV reactivation and worsened hepatic inflammation (60). A personalized treatment plan that carefully integrates antibacterial and antiviral strategies prevents treatment antagonism, thereby ensuring optimal control of both infections. Additionally, novel therapeutic approaches, including immune modulation and hepatoprotective agents, are being explored to enhance clinical outcomes in these complex cases.

### Targeting chronic hepatitis B in the absence of pyogenic liver abscesses

In patients with chronic HBV infection but without PLAs, the focus remains on comprehensive antiviral management aimed at long-term viral suppression and liver protection. Advances in HBV therapeutics have paved the way for more targeted and durable treatment strategies.

### First-line antiviral therapies: Nucleos(t)ide analogs (NAs)

The backbone of chronic HBV management lies in the use of potent NAs such as entecavir (ETV) and tenofovir (TDF). These agents effectively suppress viral replication, mitigate hepatic inflammation, and reduce the risk of cirrhosis and hepatocellular carcinoma (HCC). Their high genetic barrier to resistance ensures long-term efficacy, making them the preferred choice for most HBV patients. According to the American Association for the Study of Liver Diseases (AASLD), lifelong therapy with these agents may be required in patients with advanced liver fibrosis or immune suppression to maintain viral control and prevent disease progression.

### Interferon-based immunotherapy

Pegylated interferon (PEG-IFN) remains a viable option for select patients, offering the potential for a finite treatment course. While associated with a less favorable safety profile due to systemic immune activation, PEG-IFN can induce sustained virological responses in a subset of patients, particularly those with favorable host immune profiles.

### Cutting-edge therapeutic innovations

The landscape of HBV treatment is rapidly evolving with the emergence of novel therapeutic strategies aimed at achieving functional cure by eradicating covalently closed circular DNA (cccDNA) or modulating host immune responses. Key advancements include: Epigenetic Modulation: Targeting cccDNA silencing to reduce HBV persistence; RNA Interference (RNAi): Utilizing gene-silencing techniques to suppress HBV protein expression; CRISPR/Cas Genome Editing: Exploring precise genetic modifications to eliminate HBV reservoirs; Therapeutic Vaccines and Immune Checkpoint Inhibitors: Enhancing antiviral immune responses to facilitate viral clearance. Ongoing research and clinical trials are pivotal in refining these therapies, with the ultimate goal of achieving a functional cure for HBV and improving patient outcomes.

The presence or absence of pyogenic liver abscesses in chronic HBV-infected patients necessitates distinct yet equally strategic management approaches. When PLAs are present, a dual-pronged strategy integrating aggressive antibacterial treatment, precise abscess drainage, and optimized antiviral therapy is essential to mitigate both bacterial and viral threats. In contrast, patients without PLAs benefit from robust antiviral regimens centered on nucleos(t)ide analogs, with emerging therapeutics offering the potential for HBV eradication in the future. By harnessing personalized treatment strategies, leveraging novel therapeutic innovations, and maintaining vigilant clinical oversight, healthcare providers can significantly improve the prognosis and quality of life for patients battling chronic HBV infection, with or without the added complexity of pyogenic liver abscesses.

## Discussion

Chronic Hepatitis B Virus (HBV) infection presents substantial clinical challenges, particularly when complicated by hepatocellular carcinoma (HCC) and pyogenic liver abscesses (PLA). These coexisting conditions create a highly complex clinical landscape that demands a nuanced approach to treatment. Understanding the molecular mechanisms that underpin HBV infection and its associated complications is essential for the development of effective, personalized therapeutic strategies for these intricate scenarios (13, 14, 16, 61–63). The complexity is further compounded by the convergence of other infectious diseases, such as COVID-19, which introduce additional layers of risk and therapeutic uncertainty in managing HBV-related pathologies (64–72). Viral infections are increasing day by day and there is urgent need for centrally controlled global disease surveillance (63, 73–88). There is an urgent need to implement and scale targeted interventions, whether through financial incentives or universal vaccination programs, to improve health outcomes and reduce the burden of viral hepatitis and its complications, emphasizing the critical importance of adopting multifaceted approaches in public health strategies (89, 90).

This review offers critical insights into the molecular underpinnings of HBV persistence, with a particular focus on the covalently closed circular DNA (cccDNA), a central player in viral latency and persistence. By delving into the regulatory processes involved in cccDNA formation, maintenance, and clearance, this review provides clinicians with a deeper understanding of the persistent challenges in HBV management. The ability of cccDNA to persist in liver cells despite antiviral therapy remains a significant obstacle in achieving a functional cure, making it a focal point for research into novel therapeutic strategies.

A thorough exploration of current and emerging therapeutic approaches aimed at targeting cccDNA uncovers a diverse range of promising strategies. From epigenetic regulation to cutting-edge RNA interference technologies and CRISPR/Cas9 gene editing, several innovative interventions are being investigated to disrupt cccDNA persistence. Recent studies have shown that histone deacetylase inhibitors (HDACi) and DNA methylation modifiers offer substantial potential in mitigating cccDNA’s stability and persistence, providing new hope for overcoming one of the most formidable barriers to effective HBV treatment (7, 19). These approaches are paving the way for therapeutic advancements that may ultimately lead to more successful and durable outcomes in the fight against chronic HBV infection.

RNA interference (RNAi) therapeutics, particularly small interfering RNA (siRNA) and short hairpin RNA (shRNA), have emerged as promising strategies for selectively targeting cccDNA-derived transcripts, thereby inhibiting viral replication and reducing the risk of hepatocellular carcinoma (HCC) [Wooddell et al. (29)]. These RNA-based therapies hold significant potential in disrupting the persistence of HBV by silencing viral genes at the transcript level. Similarly, CRISPR/Cas systems are providing innovative tools to directly target and cleave HBV cccDNA, offering transformative strategies for eliminating or mutating the viral genome. These cutting-edge approaches represent a novel frontier in HBV treatment, with the potential to substantially impact the long-term management of chronic infection (34, 35).

The inclusion of HCC patients with pyogenic liver abscess (PLA) further underscores the urgent need for specialized therapeutic approaches to manage the complex interplay between HBV infection, HCC, and PLA. These conditions present unique challenges that require a multifaceted treatment strategy. The intricate relationship between HBV persistence and the development of HCC and PLA highlights the need for comprehensive, individualized care plans. By deepening the understanding of the molecular mechanisms driving HBV persistence and its association with these complications, clinicians can better tailor therapeutic interventions to address the diverse challenges presented by these co-existing conditions (55–57).

Looking ahead, future research must prioritize translating molecular insights into clinically actionable strategies that can be applied to real-world patient care. Collaborative efforts between clinicians, basic scientists, and translational researchers are crucial to bridging the gap between laboratory discoveries and clinical applications. Notably, studies examining the potential of immunomodulatory agents, such as immune checkpoint inhibitors, in conjunction with antiviral therapies show great promise in enhancing host immune responses against HBV-infected hepatocytes. Such combinatorial approaches could offer new avenues for achieving more effective and durable treatment outcomes in chronic HBV infection.

Advancements in individualized therapy for HBV, while promising, bring significant challenges to health systems, particularly regarding cost and accessibility. Precision medicine, driven by genomic profiling and targeted therapeutic regimens, enables tailored treatment plans that optimize outcomes for individual patients. These therapies hold the potential to improve the quality of life, reduce adverse effects, and address specific disease characteristics, leading to better management of chronic HBV and its complications. However, the infrastructure required to implement such cutting-edge treatments, including advanced diagnostic technologies, highly skilled healthcare providers, and continuous research and development, contributes to substantially higher costs. These expenses often make individualized therapies financially inaccessible to large segments of the population, particularly in low- and middle-income countries. Consequently, the disparity in access to such interventions exacerbates existing health inequities and places an unsustainable burden on already constrained healthcare budgets. In contrast, investment in preventive strategies offers a cost-effective and scalable solution to controlling HBV at the population level. Universal vaccination programs, for instance, have demonstrated remarkable success in reducing HBV transmission rates and the prevalence of chronic infection. Vaccination not only prevents new cases but also indirectly reduces the incidence of severe complications such as cirrhosis and hepatocellular carcinoma, which are far more expensive to treat. Public health campaigns focused on awareness, education, and early detection further enhance prevention efforts, while harm reduction strategies, such as needle exchange programs and safe injection practices, specifically target high-risk populations, curbing the spread of HBV in vulnerable groups. Prevention also carries significant long-term economic benefits. By reducing the overall burden of HBV, health systems can allocate resources more effectively, avoiding the high costs associated with managing advanced stages of the disease or complications requiring hospitalizations, liver transplants, or extended therapeutic interventions. Furthermore, robust prevention programs build resilience within health systems, enabling them to address HBV alongside other public health challenges, fostering a holistic approach to healthcare delivery. Policymakers must navigate the delicate balance between integrating individualized therapies and sustaining preventive measures. While high-cost, precision medicine treatments should remain available for patients with complex needs, the prioritization of prevention must be central to any comprehensive HBV management strategy. Investing in preventive infrastructure not only reduces future demand for expensive therapies but also ensures broader and more equitable access to healthcare. A dual approach, combining prevention with selective use of individualized therapies, is essential for maintaining manageable healthcare costs while delivering optimal outcomes. This strategy underscores the importance of prevention as the foundation of sustainable HBV management, ensuring that healthcare systems can effectively address the disease without becoming financially overburdened by ever-increasing reliance on costly novel therapies.

## Conclusion

Current study underscores the urgent need to understand the molecular intricacies of HBV infection and its associated complications to unlock transformative advances in patient care. By delving into the complex mechanisms of viral persistence, immune evasion, and disease progression, particularly in the context of HCC and PLA, we pave the way for the development of more precise and personalized therapeutic strategies. The path forward lies in the continued collaboration between clinicians, researchers, and scientists, fostering the integration of groundbreaking discoveries into clinical practice. Future research must focus on leveraging innovative approaches, such as gene editing, precision medicine, and immune modulation, to target viral reservoirs like cccDNA and ultimately eradicate HBV. The take-home message is clear: through a deeper understanding of HBV’s molecular foundation, we have the potential to revolutionize treatment strategies, offering patients not just better outcomes, but a future where HBV is no longer a chronic threat but a manageable condition with long-term remission.
